# Cancer and Neurodegeneration: Between the Devil and the Deep Blue Sea

**DOI:** 10.1371/journal.pgen.1001257

**Published:** 2010-12-23

**Authors:** Hélène Plun-Favreau, Patrick A. Lewis, John Hardy, L. Miguel Martins, Nicholas W. Wood

**Affiliations:** 1 Department of Molecular Neuroscience, UCL Institute of Neurology, London, United Kingdom; 2 Cell Death Regulation Laboratory, MRC Toxicology Unit, Leicester, United Kingdom; University of Washington, United States of America

## Abstract

Cancer and neurodegeneration are often thought of as disease mechanisms at opposite ends of a spectrum; one due to enhanced resistance to cell death and the other due to premature cell death. There is now accumulating evidence to link these two disparate processes. An increasing number of genetic studies add weight to epidemiological evidence suggesting that sufferers of a neurodegenerative disorder have a reduced incidence for most cancers, but an increased risk for other cancers. Many of the genes associated with either cancer and/or neurodegeneration play a central role in cell cycle control, DNA repair, and kinase signalling. However, the links between these two families of diseases remain to be proven. In this review, we discuss recent and sometimes as yet incomplete genetic discoveries that highlight the overlap of molecular pathways implicated in cancer and neurodegeneration.

## Introduction: Epidemiological Data

At first glance, cancer and neurodegeneration seem to have little in common. Although neurodegeneration results in the death of post-mitotic neurons, cancer cells are characterised by an enhanced resistance to cell death. However, the more we learn about the molecular genetics and cell biology of cancer and neurodegeneration, the greater the overlap between these disorders appears. Many of the recent findings in both fields offer new avenues of study for these two age-related conditions, addressing an urgent need for therapeutic options, especially for patients with advanced disease.

Many epidemiological studies have linked cancer and neurodegenerative disorders. A growing body of evidence suggests an inverse correlation between the risk of developing cancer and a neurodegenerative disorder, in particular Parkinson's disease (PD). Several case-control and cohort studies have reported a reduced risk of almost all cancers, both smoking-related and non-smoking-related, among individuals with PD [1]. The exception to this is a suggestion of an increased risk of malignant melanoma associated with a PD diagnosis [2]–[7]. Additional work has also identified a possible association between melanoma and amyotrophic lateral sclerosis (ALS), a form of motor neuron disease (MND) [8], [9]. Nevertheless, a recent study showed no significant association between cancer and either MND or multiple sclerosis [10], in contrast to previous reports [11]–[17]. Fewer data are available linking cancer and either Alzheimer's disease (AD) or Huntington's disease (HD). It has been shown that, after adjustment for age, a diagnosis of AD was associated with a 60% reduced risk of cancer, and a history of cancer was associated with a 30% reduced risk of AD [18], [19]. Concerning HD, a lower incidence of cancer was observed among patients with the disease [20].

There is, of course, a difference between association and causality, and it has been proposed that the association between PD and skin cancer could be linked to treatment for PD (e.g., Levodopa therapy) rather than with the disease itself. However, recent reviews of the evidence do not support such a causal association [21], [22]. Additionally, it has been suggested that the decreased incidence of cancer in patients with PD is linked to the negative association between PD and smoking [23]. Although this may account for much of the risk reduction regarding smoking-related cancers, it fails to explain the decrease of non-smoking-related cancers.

The origins of the association and interplay between cancer and neurodegeneration are still a matter of debate, but increasing evidence suggests that new discoveries in genetics of these two conditions may help scientists solve the cancer–neurodegeneration enigma in the coming decade. A number of studies show that the genes causing neurodegeneration are often mutated or abnormally expressed in cancer. In the following sections we use a series of examples to illustrate the emerging genetic evidence linking cancer and neurodegeneration. We discuss whether genes that predispose to cancer also cause neurodegeneration and vice versa. Moreover, we review the genomic means of unravelling the emerging molecular pathways linking cancer and neurodegeneration.

## Proven Genetic Factors Implicated in Both Cancer and Neurodegeneration: The *ATM* Gene

The vast majority of cancers and neurodegenerative disorders in the general population are sporadic in nature but a small proportion of these (5%–10%) are inherited in a Mendelian fashion. The search for the genes responsible for these familial forms of disease has been dominated over the last 20 years by the identification of genes that cause monogenic forms of disease. Such mutations have been discovered predominantly through linkage studies, which typically find high penetrance, but rare, genetic variants. Several genes have been unambiguously shown to cause rare familial forms of neurodegeneration [24], [25] and cancer syndromes [26]. *AT-mutated* (*ATM*) provides the closest genetic link between neurodegeneration and cancer thus far.

Ataxia-telangiectasia (AT) is a rare neurodegenerative autosomal recessive disease characterised by chromosomal instability, immunodeficiency, and a predisposition to cancer. This disease is caused by mutations in the *ATM* gene that leads to a total loss of the ATM protein kinase, which is part of the phosphatidylinositol-3 kinase (PI3K) superfamily, and plays a central role in cell division and DNA repair. Mutations in other DNA repair genes have been shown to cause both cancer and neurodegeneration [27]. Whether DNA repair is a causal link between cancer and neurodegeneration remains, however, to be proven. Nearly 40% of *ATM* homozygotes will develop cancer, usually childhood leukaemia or lymphoma [28]–[30]. Strikingly, *ATM*-heterozygote germline mutations were also shown to contribute to breast cancer susceptibility [31], [32]. It is noteworthy that the kinase encoded by the *ATM* gene is a prominent activator of p53 [27], a key tumour suppressor protein mutated and inactivated in approximately 50% of human cancers [33]–[37]. *ATM* is a good example of a gene that functions as a tumour suppressor but whose inactivation also leads to neuronal loss when the mutations are in the germline [38], [39].

## Proven Genetic Factors Implicated in Neurodegeneration and Putatively Implicated in Cancer: The *PARK2* Gene

The *PARK2* gene encodes parkin, an E3 ubiquitin ligase. This gene is the most commonly mutated gene in autosomal recessive PD [40]. *PARK2* was a putative candidate for a tumour suppressor gene [41]–[44], with identified whole exon deletions and duplications of this gene in ovarian and other cancers supporting this hypothesis [42], [45]. More recently, chromosomal microarray analysis was used to identify *PARK2* somatic mutations and intragenic deletions in glioblastoma, colon cancer, and lung cancer [46]. This suggests that while germline mutations in PARK2 cause PD, somatic mutations in PARK2 contribute to cancer. However, *PARK2* is a very large gene prone to deletions and mutations, and whether somatic mutations in *parkin* are primarily involved in the tumour development remains to be confirmed. Homozygous or compound heterozygous *PARK2* mutations unambiguously cause PD [40]. Several lines of evidence suggest that heterozygous *PARK2* mutations also have a role in the development of parkinsonism, although this is a matter of debate [47], [48]. Notably, only a few alterations identified in cancer were homozygous, most being heterozygotes. Strikingly, these mutations sufficiently altered parkin's ability to promote tumour growth. Therefore, these data suggest that, in cancer, *PARK2* may act in a haploinsufficient manner.

Interestingly, *PARK2* and *ATM* mutations in cancer sometimes occur at the exact same residue, causing neuronal degeneration [30], [46], [49]. This observation supports the idea that not only similar molecules but also similar genetic mutations within the same molecule can have very different effects, depending on the type of cell in which they occur: a dividing cell in cancer or a post-mitotic neuron in neurodegeneration. Notably, neurons are not the only post-mitotic cells, and yet they are the main cell type affected in neurodegenerative disorders. Rather than mitosis on its own, a combination of neuronal functions is therefore likely to explain the link between cancer and neurodegeneration disorders.

It is not yet clear whether the germline pathogenic mutations in the *PARK* genes can also increase the risk for cancer. One way to answer this question would be to compare the frequency of tumours in PD patients carrying heterozygote, compound heterozygote, or homozygote mutations in the *PARK* genes to that of idiopathic patients and controls without *PARK* mutations. However, this kind of study design is difficult to achieve in an epidemiologically robust fashion. It would require a very large number of cases and other epidemiological data as well as detailed family history and risk factor assessment.

A number of somatic mutations in two other genes unequivocally linked to PD, namely *PINK1* and *LRRK2* [50]–[52], both of which encode protein kinases, were identified in tissue samples from patients with various tumours [53]. The dysregulation of kinases in cancer and neurodegeneration is discussed in more detail later in the text (A Catalogue Of Somatic Mutations In Cancer can be accessed via the Wellcome Trust Sanger Institute COSMIC Web site at http://www.sanger.ac.uk/genetics/CGP/cosmic/). The *PINK1* and *LRRK2* somatic mutations identified in cancer were all heterozygous and their pathological effect remains to be determined. The prevalence of *LRRK2* G2019S (the most common genetic determinant of PD) is not increased in patients with melanoma [7], [54], but a recent study showed an almost 3-fold increased risk of non-skin cancers in *LRRK2* G2019S mutation carriers [55]. Moreover, of the 18 known mutation carriers of a large family with *LRRK2* R1441C parkinsonism, four had colon cancer [56]. Nevertheless, further studies will be required to ascertain whether the association between *LRRK2* parkinsonism and cancer is real or coincidental. Given the frequency of the G2019S mutation in Ashkenazi Jews and Arab Berbers with PD, it should be possible to conduct large epidemiological studies looking at cancer incidence in these families [57].

It is noteworthy that the monogenic forms of neurodegeneration and cancer are, on the whole, very rare. While most of what we know about the molecular background of idiopathic diseases is based on information gleaned from the study of rare familial forms of these disorders, one cannot readily assume that any information learnt from the Mendelian forms of a disease can enlighten us about the idiopathic forms of this disease. In light of this, extending a link that might exist between monogenic disorders to the sporadic forms of cancer and neurodegeneration should be attempted with caution.

## Proven Genetic Factors Implicated in Cancer and Putatively Implicated in Neurodegeneration

It is not always the case that cancers are less common in patients with neurodegenerative disease. This is exemplified by melanoma, which has a recognised increased incidence in PD patients. A positive family history is a strongly associated risk factor for melanoma [58]–[62], and approximately 50% of affected families have mutations in one of the three following genes: cyclin-dependent kinase inhibitor 2A (*CDKN2A*), alternate reading frame (*ARF*), and cyclin-dependent kinase 4 (*CDK4*). These mutations, identified through linkage studies, are inherited in an autosomal dominant manner and have a high penetrance. High-frequency alleles with small effects on melanoma risk have also been identified in a number of genes, including *MC1R* (Melanocortin 1 Receptor) and *TYR* (tyrosinase). Moreover, an approximately 2-fold increase in the risk of PD was reported among individuals who reported a family history of melanoma compared with individuals without such a family history. The significant association was independent of several known risk factors for PD, including smoking [63]. No significant associations were observed between a family history of several other common cancers and PD risk [64], suggesting the existence of common genetic determinants between PD and melanoma. There remains, however, the possibility that another unknown environmental factor could contribute to the observed association between a family history of melanoma and PD risk. Other genes, such as the CDKs, for which an increased expression or dysregulation has been observed in melanomas [65] and PD [66], [67], could also play a role in the observed association.

Two genome-wide association studies (GWAS) have recently been performed in melanoma and melanocytic nevi [68]–[70]. One study replicated two previously suggested associations with the disease, *MC1R* and *TYR*. In addition to hits near these two genes, a locus flanking the familial melanoma susceptibility locus *CDKN2A* was identified. The second study demonstrated that methylthioadenosine phosphorylase (*MTAP*), a gene adjacent to *CDKN2A*, and another locus encompassing *PLA2G6* (a member of the phospholipase A2 gene family) both showed an association with melanoma risk. Interestingly, mutations in the gene encoding the phospholipase PLA2G6 can cause parkinsonism [71]. PLA2G6 is also associated with lung cancer susceptibility [72]. The combination of these accumulating epidemiologic and genetic linkages between melanoma and PD suggest a need for more mechanistic/biological work in this area.

Notably, no major known cancer gene was among the combination of genetic variants identified as risk factors for neurodegenerative disorders. In fact, recent studies from GWAS of AD and PD have mainly identified genes principally implicated in protein accumulation and the complement cascade of the immune system [73].

## Post-Translational Modifications—Strongly Implicated in Cancer, with an Emerging Role in Neurodegeneration?

Post-translational modifications also play a role in the association between cancer and neurodegeneration. For example, protein alterations that predispose the cell toward cell death might lead to a decreased risk of cancer and an increased risk of neurodegeneration, whereas conditions that favour cell growth might lead to an increased risk of cancer and a decreased risk in neurodegeneration [74]–[77]. Indeed, the same molecules are often used for different purposes in the control of cell division, cell differentiation, and cell death. Depending on whether the cell is an actively dividing or a post-mitotic neuron, responses to alterations in these molecules and pathways may differ, ultimately leading to either cancer or neurodegeneration (for a comprehensive overview of the genes implicated in neurodegeneration and cancer, see Table 1).

**Table 1:** Genetic determinants at the interface of cancer and neurodegeneration.

Gene	Function	Role in Neurodegeneration	Role in Cancer
**α-synuclein (PARK1/4)**	Unclear	Gain of function leads to PD [104]. Main component of Lewy bodies in PD [105].	α-synuclein is aberrantly expressed and methylated in cancer [106].
**PINK1 (PARK6)**	Kinase	Loss of function leads to PD [50]. Loss of PINK1 functions leads to mitochondrial deficits.	Somatic mutations in cancer (COSMIC Web site). Tumour suppressor? Induced by PTEN [92].
**DJ-1 (PARK7)**	Unclear	Loss of function leads to PD [87]. DJ-1 might act as a neuroprotective oxidative stress sensor.	Oncogene [86]. Regulates negatively PTEN. Over-expression in several tumours.
**LRRK2 (PARK8)**	Kinase, GTPase	Gain of function leads to PD [51], [52]. Enzymatic activities thought to play key role in disease [105].	Somatic mutations in cancer (COSMIC Web site). Oncogene?
**ATP13A2 (PARK9)**	ATPase	Loss of function leads to PD [107]. May alter autophagic lysosomal function.	ALP plays an important role in cancer.
**PLA2G6 (PARK14?)**	Phospholipase A2	Mutations lead to infantile neuroaxonal dystrophy (INAD), idiopathic neurodegeneration with brain iron accumulation (NBIA) and dystonia-parkinsonism [71].	PLA2G6 was identified as a risk factor for melanoma [69].
**Tau (MAPT)**	Microtubule-associated protein	Mutations in Tau lead to AD and FTDP-17 [105], [108] Tau is the major component of neurofibrillary tangles in AD.	Reduced expression in several tumours.
**APP/PS1,2**	Unclear	Gain of function leads to AD type [109]. Mutations in APP and the presenilins increases production of Aβ, which is the main component of senile plaques in AD.	APP is overexpressed in acute myeloid leukemia patients with complex karyotypes [110].
**SOD1**	Superoxide dismutase	Gain of function leads to ALS. Mutations thought to cause cell death via aggregation and oxidative damage [111], [112].	Role in breast cancer? [113]
**Huntingtin**	Unclear	Gain of function leads to HD [114].	
***Parkin (PARK2)***	E3 ubiquitin ligase	Loss of function leads to PD [40]. Parkin enzymatic activity is thought to play a key role in disease. Loss of parkin function leads to mitochondrial deficits [115], [116].	Tumour suppressor [46].
***ATM***	Kinase (PI3K)	Mutations in the *ATM* gene cause ataxia-telangiectasia [30]. ATM inactivation leads to cerebellar neuron loss.	Tumour suppressor. *ATM* mutations carriers at increased risk of developing cancer, especially breast cancer. Role in cell cycle and DNA damage.
***CDK5***	Kinase	CDK5 can phosphorylate Tau [117] and parkin [118]. Also is associated with AD [95].	Somatic mutations in cancer.
*p53*	Transcription factor	Functional link between p53 and parkin, Ab and APP [83].	Tumour suppressor [33].
*PTEN*	Phosphatase	Functional link between PTEN and PINK1, parkin and DJ-1 [119].	Tumour suppressor, mutated in sporadic and inherited tumours [120].
*mTOR*	Kinase	May play a role in neurodegeneration through inhibition of autophagy.	Autophagy can be both oncogenic as well as tumour suppressive.
*TSC1/TSC2*	Vesicular transport	May play a role in neurodegeneration through mTOR-dependant autophagy.	Tumour suppressors [121].

Common factors and overlapping pathways can be identified in the progression of both cancer and neurodegeneration. A number of molecules genetically associated with these diseases are kinases and/or play a role in apoptosis, cell cycle, and DNA repair. Protein degradation pathways are often disturbed in both cancer and neurodegeneration. Mitochondrial dysfunction and oxidative stress are also shown to cause both diseases. Finally, the autophagic lysosomal pathway is increasingly recognised as playing a major role in the physiopathological mechanisms associated with both the disorders. Importantly, all these processes are regulated during aging, the first risk factor for both cancer and neurodegeneration.

**In bold—Strong genetic association with neurodegeneration.**

*In italic—Strong genetic association with cancer.*

***In bold and in italic—Strong genetic association with both cancer and neurodegeneration***.

Many proteins when abnormally expressed or aberrantly regulated have been linked to cancer or neurodegeneration; in particular, proteins implicated in cell cycle regulation [75]. For example, many human cancers have lost the function of p53, a key tumour suppressor transcription factor playing an important role in cell cycle arrest in response to DNA damage and apoptosis [33]–[37]. Increasing evidence supports the contribution of transcriptional inhibition to neurotoxicity of DNA damage [78]. Interestingly, p53 is associated with several neurodegenerative disorders, including HD, AD, and PD [35], [37]. P53 protein can regulate *huntingtin* (*htt*) expression at transcriptional level [79]. Moreover, p53 provides strong protection from neurotoxicity associated with the mutant htt with expanded polyglutamine in HD fly and mouse models [80]. The PD-associated protein parkin can repress p53 transcriptional activity that is impaired by the *PARK2* mutations associated with PD [81], [82]. Finally, p53 regulates and is regulated by AD-associated proteins such as the members of the γ-secretase complex [83]. A recent review discusses the role of p53 as a potential candidate that may explain the inverse association between AD and cancer [84]. It would be interesting to determine whether patients with Li-Fraumeni syndrome, characterised by germline mutations in the *p53* gene [85], have an altered risk for neurodegeneration. Cancer-related proteins can cause neurodegeneration when abnormally expressed or regulated and the opposite is also true. A number of genes associated with neurodegeneration were investigated in cancer research before their role in neurodegeneration was identified, but whether these genes are true oncogenes or tumour suppressors remains to be proven. For example, *DJ-1* was identified as an oncogene before it was linked to autosomal recessive PD [86], [87]. This gene was initially cloned as a cMyc interactor. It is expressed at high levels in lung and prostate cancer biopsies and in the sera of breast cancer patients [88]–[90]. DJ-1 was shown to suppress the function of the tumour suppressor PTEN [91], a gene shown to induce *PINK1* when overexpressed [92]. However, DJ-1 showed a weak transforming activity by itself, throwing into doubt its oncogenic function [86].

Protein kinases, when abnormally expressed or dysregulated, can lead to cancer. Because of the key apical role of kinases in the control of key signal transduction networks that impact normal cellular physiology and pathological conditions, the development of small molecule kinase inhibitors as potential cancer therapeutics is an area of intense research. A subset of these agents target CDK activity. Interest in the therapeutic potential of CDK inhibitors has expanded to include neurodegenerative diseases [93]. Specifically, there is growing evidence suggesting that CDK5, an important modulator of neuronal activity and a critical player in a number of cancers, is involved in various physiological roles within the central nervous system and a number of neurodegenerative disorders such as AD, ALS, HD, and PD [94]. Interestingly, variations in the *CDK5* gene are associated with AD [95]. Finally, as a result of their putative kinase function, PINK1 and LRRK2 are attractive potential targets in the treatment of PD and cancer even though their potential influence in tumour growth remains mostly indirect and suggestive thus far (see Table 1; [1], [96], [97]).

## Challenges for the Future

Although many epidemiologic studies have found a relationship between cancer and neurodegeneration, in particular in PD, the results have been inconsistent. Variations in the design, methods, and quality of the studies on cancer risk among patients with PD have made it difficult to ascertain the link between the two disorders. In the next section, we discuss the means of exploring this link in order to accelerate progress in the next few years. Our understanding of the control of signalling pathways is further advanced in cancer studies compared to neurodegeneration. As a result, many small molecule inhibitors, such as histone deacetylase inhibitors and kinase inhibitors, have been approved as anticancer agents or are currently being tested in clinical trials [98]. Thus, discoveries in cancer research are likely to provide a solid base upon which scientists will study the pathophysiology of neurodegenerative diseases.

The results of the many epidemiologic studies that have found patients with a neurodegenerative disease to be associated with a modified incidence of cancer have varied in their consistency. Diversity in the design and quality of the studies exploring cancer risk in patients with neurodegenerative disease has made it difficult to confirm the relationship between the two diseases with certainty. The GWAS approach has effected a step change in human genetic research by linking a number of variants to complex diseases. Each variant robustly linked to a disease offers a possible route to unravelling the molecular pathways associated with the disease. GWAS have been performed for most cancers and neurodegenerative disorders; a catalog of published GWAS is available online at http://www.genome.gov/. However, the results of GWAS have also been variable [73], and it is likely that much larger epidemiologic and genetic studies and meta-analysis will be required to determine if there is a real association between cancer and neurodegeneration. A quantitative analysis of several independent studies has confirmed the overall lower cancer risk ratio among patients with PD [99].

Although the variants that have been identified thus far confer only a small risk of the disease, identifying additional variants that contribute to the pathogenesis of the disease is likely to help the scientific community to move forward in understanding the link between these two disorders. With this in mind, a second generation of GWAS will be performed using new chips targeting variants throughout the genome at even lower frequencies. Additionally, as sequencing technology becomes cheaper, an explosion of targeted gene-sequencing studies looking for rarer risk variants is to be expected. The use of approaches such as array-based comparative genomic hybridisation, high-throughput sequencing, and transcriptome analysis has already enabled the identification of common variants for cancer and neurodegeneration, for example *PARK2* in cancer [46].

The next generation of sequencing is also likely to help with the understanding of the link between cancer and neurodegeneration. Exome sequencing may represent only an intermediary step before whole-genome sequencing becomes widely available. However, this technology may still be able to shed light on important coding mutations in these disorders. It is important to note that this approach can miss potentially important non-coding changes (e.g., regulatory regions or miRNAS), which will require the systematic approach offered by whole-genome sequencing. Some major cancer genome screening projects aim to eventually sequence the full genomes of thousands of tumour samples and those of people from whom the tumours were taken. Currently, most laboratories investigating these diseases are carrying out exome sequencing, although whole-genome sequences of a patient with acute myeloid leukaemia have already been obtained [100].

Finally, it is becoming increasingly clear that a multitude of complex and interconnected epigenetic modifications such as miRNAs, DNA acetylation, and DNA methylation can conspire with genetic alterations in disease pathogenesis [101]. As a result, methodologies like genome-wide promoter DNA methylation profiling could reveal specific patterns that are associated with the disease [102].

## Conclusion

**Figure 1:**
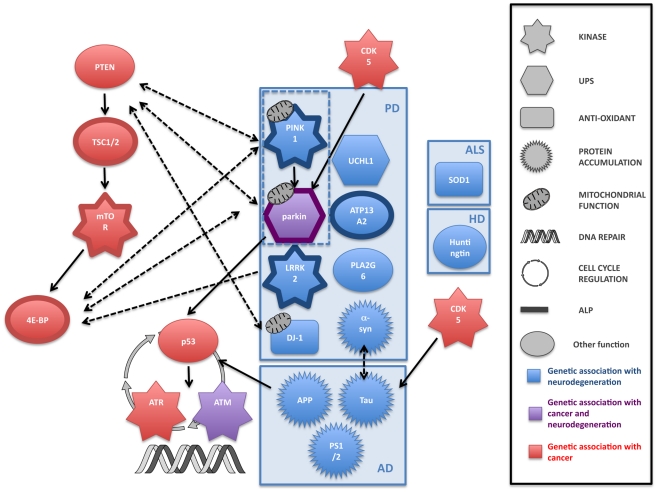
Common pathways to cancer and neurodegeneration? An illustration of some of the genes that are linked to cancer and neurodegeneration, and the crosstalk plus overlap between them. Although the links between genes involved in the individual disorders themselves are not yet completely clear (for example, there is evidence that there may be several parallel pathways leading to cell loss in the *substantia nigra* and the clinical symptom of parkinsonism), there is an intriguing picture emerging of fundamental links between cell proliferation and cell death. ALP, autophagy-lysosome pathway; UPS: ubiquitin-proteasome system.

Both cancer and neurodegeneration are thought to be the result of the interaction of genetic and environmental factors [103]. Age is the single most important risk factor for both cancer and neurodegeneration and, although the exact mechanisms of aging are not yet completely defined, age is likely to play an important role in the link between the two disorders. Both cancer and neurodegeneration are also characterised by the contribution of the inheritance of mutated genes. Research showing that cancer and neurodegenerative disorders share some of the same genes and molecular mechanisms strengthens the idea that individuals affected by a neurodegenerative disease may have a decreased risk of some cancers. Despite a number of intriguing pointers, little is known about the genetic association between cancer and neurodegeneration. Although a large number of genes have been implicated in the genesis of cancer and neurodegeneration, only two, *parkin* and *ATM*, have been shown to strongly overlap (Figure 1). Given the large number of signalling molecules that crosstalk in multiple pathways, one cannot exclude that these overlaps could be coincidental. Further, large genetic and epidemiological studies looking at cancer incidence in the population afflicted with neurodegenerative disease (and vice versa) will be required to find putative new genes at the interface of the two diseases and to ascertain that the genetic link between these two disorders is not coincidental. Unravelling the precise molecular processes that may be involved in both disorders is likely to be enlightening. Most degenerative diseases of the brain are incurable and the study of tissue from the brains of people with significant neurodegeneration should be approached with caution because the neuronal cells that are dysregulated and likely to be most informative are already dead. However, cancer research has been extremely prolific over the past two decades, and one could imagine that research in neurodegeneration will benefit from breakthrough studies in cancer. Therefore, the extensive therapeutic developments in cancer research may allow the identification of prognostic markers for cancer and neurodegeneration that could result in improved treatments for both disorders.
